# Locking plate combined with titanium cable for Neer type II distal clavicle fractures

**DOI:** 10.1186/s12891-021-04137-4

**Published:** 2021-03-12

**Authors:** Fan Zhang, Qiang Fu, Yongchuan Li, Nan Lu, Aimin Chen, Liangyu Zhao

**Affiliations:** Department of Orthopaedics, Changzheng Hospital, Second Military Medical University, 415# Fengyang Road, Huangpu District,, 200003 Shanghai, China

**Keywords:** Distal clavicle fractures, Neer type II, Combined surgical strategy, Locking plate, Titanium cable, Coracoclavicular stabilization

## Abstract

**Background:**

There is controversy about the optimal strategy treatment of unstable distal clavicle fractures. We explored use of a surgical strategy: combination of a distal clavicle locking plate and a titanium cable. The purpose of this retrospective case series study was to evaluate the clinical outcomes of this new strategy in treating Neer type II distal clavicle fractures.

**Methods:**

Twenty-one patients with Neer type II distal clavicle fracture were treated with a distal clavicle locking plate and a titanium cable. All patients were followed for at least one year. We retrospectively collected and reviewed follow-up data. The clinical outcomes were assessed for union rate, functional score, and complication rate.

**Results:**

All patients achieved bony union within 6 months. The average follow-up period was 20.6 months (range12–48 months). The mean Constant score at one-year follow-up was 94.6 ± 3.3 (range 84–98), and the mean UCLA (University of California, Los Angeles) score was 33.3 ± 2.6 (range 27–35). Only one complication (wound infection) was observed during follow-up. Two patients received another operation for implant removal due to local irritation.

**Conclusions:**

Use of a distal clavicle locking plate in combination with a titanium cable for treatment of Neer type II fractures results in high union rate, a low complication rate, and good shoulder function. This combined surgical strategy can be considered an effective method for treating unstable distal clavicle fractures.

## Background

Clavicle fractures are common, accounting for 2.5–5 % of all fractures in adults and 10–15 % in children. About 10–28 % of these fractures involve the distal clavicle [1, 2]. Neer has classified distal clavicle fractures into two basic types depending on the continuity of the coracoclavicular ligament [3], and type III is a special type with intra-articular extension according to the modified Neer’s classification [4]. For Neer type II distal clavicle fracture, coracoclavicular ligaments detach from the medial fragment, and consequently these fractures are unstable and displace significantly. As conservative treatment has been reported to result in relatively high nonunion and delayed union rates [5], surgical treatment has become the preferred choice of orthopedic surgeons in dealing with Neer type II distal clavicle fractures [4, 6].

To date, multiple operative techniques have been used in treating Neer type II distal clavicle fractures, yet no single technique has been generally accepted as the gold standard treatment [1]. Recently, use of a locking plate implant with a specific contoured design has been introduced for distal clavicle fractures [7–9]. These implants can improve the stability of the fixation by enabling more locking screws to be inserted into the distal fragment. However, without reconstruction of coracoclavicular ligaments, this technique may not be able to oppose the vertical directed forces, so it can led to complications [2, 10]. Thus, several studies have proposed the combined use of a locking plate and a coracoclavicular stabilization technique for treating unstable distal clavicle fractures [11–14].

**Fig. 1:**
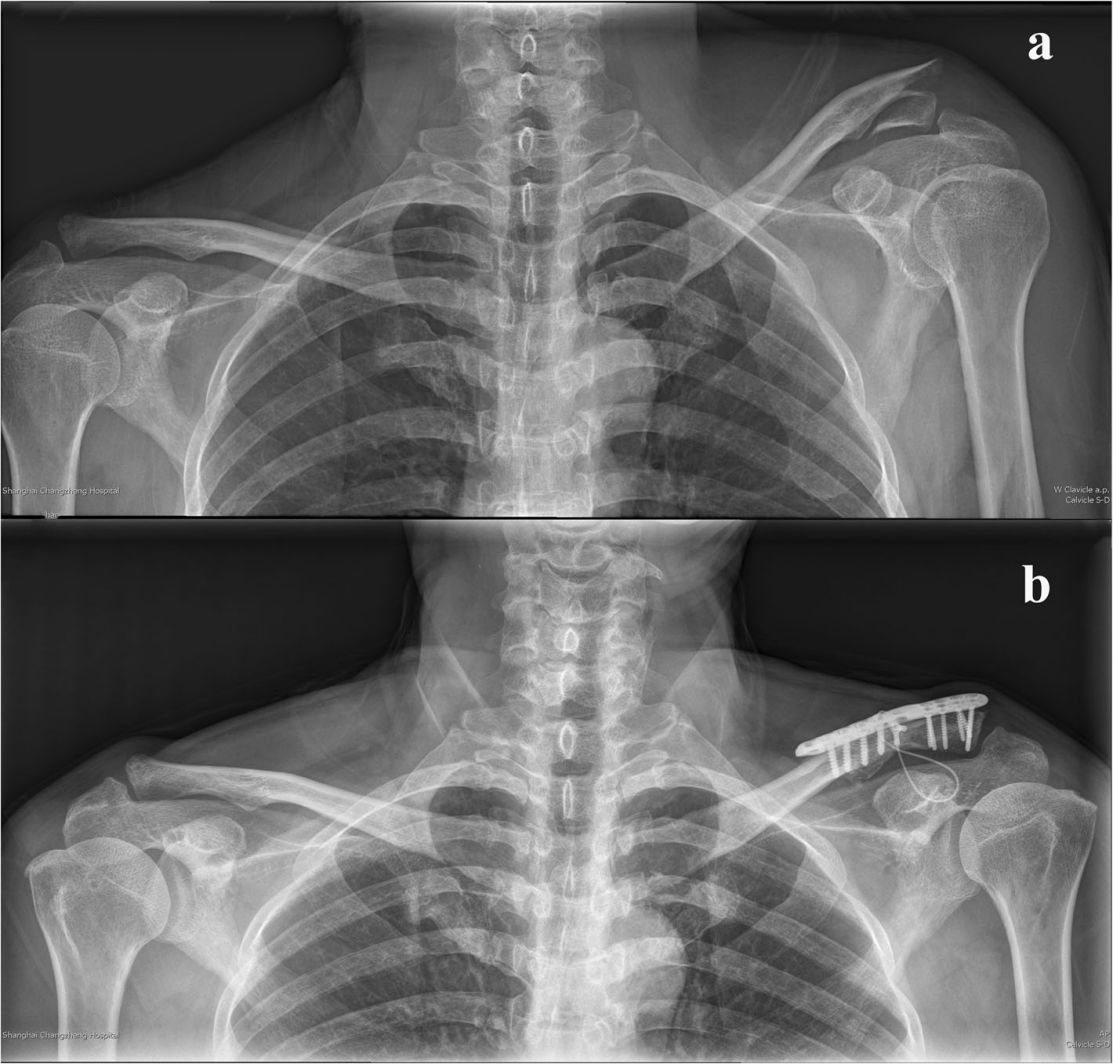
**a** Pre-operative radiograph shows a displaced distal clavicle fracture with an increased coracoclavicular distance. **b** Post-operative radiograph shows an anatomical reduction

Titanium cables are widely used in orthopedic surgeries, and this technique for coracoclavicular stabilization has already been used successfully for the treatment of acromioclavicular (AC) joint dislocation [15]. However, the use of a titanium cable alone may be insufficient for dealing with unstable distal clavicle fractures. So, we proposed a new combined surgical strategy. Over the past several years, we have used a titanium cable combined with a distal clavicle locking plate to treat Neer type II distal clavicle fractures (Fig. 1). The aim of the present retrospective case series study was to assess clinical outcomes after this surgery; we evaluated the hypothesis that this combined surgical strategy may be an effective method for the treatment of unstable distal clavicle fractures.

## Methods

### Study population

This was a retrospective case series study that was approved by the Medical Ethics Committee of our hospital. From January 2012 to March 2019, 25 patients with Neer type II distal clavicle fracture were treated with a combined surgical technique (using a locking plate combined with a titanium cable) in our hospital (a Level 1 trauma center). The inclusion criteria were patients with acute fractures (within 3 weeks of injury) and patients who aged at least 18 years. Our exclusion criteria included pathological fracture, old fracture, open fracture, multiple fracture, concomitant plexus, or nerve injuries. We also excluded patients with existing severe systemic diseases as well. We also excluded patients who were lost to follow-up. On admission, X-rays of the injured shoulder were obtained for all patients. To facilitate comparison of displacement status, radiographs of the contralateral shoulder were also obtained. All patients were treated by the same team and gave written informed consent prior to operation.

### Surgical technique

**Fig. 2:**
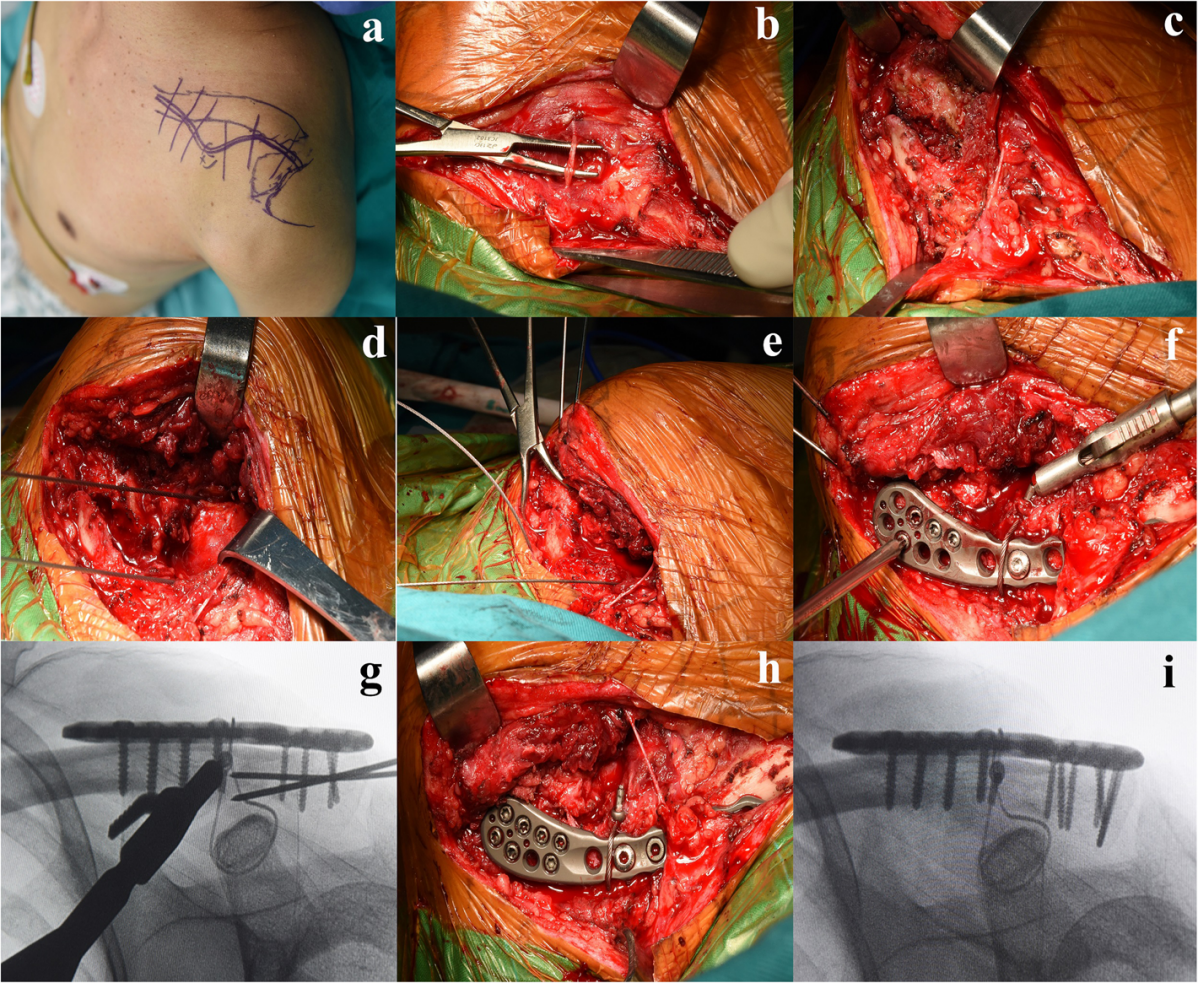
Illustration of the surgical procedures

All surgeries were performed under general anesthesia with patients placed in the beach-chair position. Before skin preparation and draping, coracoid process and acromion were marked on the skin with a marker pen. An 8-10 cm curved incision was made over the distal clavicle to the acromion; we then dissected directly to the clavicle. And the coracoid process was carefully exposed by separating the deltoid fibers. Once the medial and lateral fragment were identified, we curetted hematoma and soft tissue between the fracture ends. Usually, it was not difficult to reduce the fracture; we reduced the fracture manually or by using a pointed reduction forceps. Then, one or two Kirschner wires were inserted from the lateral fragment to the shaft of the clavicle to provisionally immobilize the fracture. In cases with comminuted fragments, the insertion of Kirschner wires across the acromioclavicular joint was employed as an alternative method. Once the fracture reduction had been confirmed by fluoroscopy, a distal clavicle anatomical locking plate (ITS, Austria) was secured to the distal clavicle. This locking plate enabled the insertion of six-to-eight 2.4mm locking screws into the lateral fragment. In order to reinforce vertical stability, a multistrand titanium cable (Medtronic, USA) was used as a complement. We applied the titanium cable around the base of the coracoid process and across the superior surface of the plate or passed it through holes that were pre-drilled in the clavicle. Then the titanium cable loop was tightened and secured with a tensioner instrument to maintain the vertical stability of the fracture. A technical detail here should be emphasized: when using a tensioner instrument to tighten the titanium cable loop, the coracoclavicular distance should not be excessively reduced, because it may limit the normal subtle motion of the acromioclavicular joint. Fluoroscopy was performed again to confirm the reduction and the placement of implants. The surgical procedures are presented in Fig. 2.

### Postoperative care

As this surgical technique provides stable fixation for the fracture in both the vertical and horizontal directions, no shoulder sling was used routinely. Only two patients (both of whom were older than 65 years) were placed into a shoulder sling (for 4weeks postoperatively, owing to their concomitant osteoporosis). Passive exercises were started within three days after surgery. We typically recommended a passive pendulum exercise to the patients as the postoperative daily training program. Four weeks after the operation, active motion of the injured shoulder with no weight bearing was encouraged. Full range of motion without resistance was allowed from 6 to 8 weeks after the operation, and weight lifting started from postoperative weeks 8–10. Once osseous union was confirmed on radiographs, unrestricted use of the injured extremity was encouraged.

### Follow‐up and outcome evaluation

All of the patients included in our study had a follow-up of at least one year. The mean follow-up was 21 months, ranging from 12 to 48 months. Follow-up evaluations of clinical outcomes and complications were performed at 6 weeks, 12 weeks, 6 months, and 12 months after surgery and annually thereafter. Radiographs were obtained at each visit to assess fracture healing, loss of reduction, and migration of implants. The range of motion was recorded at each follow-up, and shoulder function was evaluated using the Constant score and the modified UCLA score. The visual analog scale (VAS) was evaluated as well. We retrospectively reviewed all radiographs and medical records as well as complications.Data were collected independently by two reviewers prior to further data analysis using SPSS software (version 22.0, IBM, USA).

## Results

A total of 21 cases with at least one year of follow-up were included in this study. There were 15 males and 6 females, with a mean age of 43.1 years (range18–68 years). Over a half of the patients conditions resulted from falling down (13 patients, 61.9 %); the remainder were caused by traffic accidents (5 bicycle accidents and 3 motor vehicle accidents). The average duration between injury and surgery was 2.4 days (range1–7 days). Operative procedures took a mean time of 86 min (range70–110 min), with an average intraoperative blood loss of 203.3 ml (range130–330 ml). All patients achieved osseous union within half a year, with 13 achieving osseous union within three months (accounting for 61.9 %). The average follow-up period was 20.6 months (range12–48 months). The detailed patient demographics and clinical data are summarized in Table 1.

**Table 1:** Patient demographics and clinical data

Patient#	Sex	Age (years)	Side	Injury Mechanism	Systemic Disease	Time to Surgery (days)	Operation Duration (mins)	Blood Loss (ml)	Follow Up (months)	Complications
1	Male	34	Left	Vehicle Accident	None	6	90	280	24	None
2	Male	31	Right	Fall Down	None	1	80	200	12	None
3	Female	62	Left	Fall Down	None	2	100	330	12	None
4	Male	57	Left	Fall Down	None	1	95	180	24	None
5	Female	18	Left	Vehicle Accident	None	3	85	150	12	None
6	Male	46	Left	Bicycle Accident	None	2	90	200	24	None
7	Female	50	Right	Bicycle Accident	None	1	85	220	24	None
8	Male	68	Left	Fall Down	Hypertension	2	110	300	48	None
9	Male	32	Left	Fall Down	None	1	75	150	12	None
10	Female	65	Right	Fall Down	None	5	80	180	24	None
11	Male	55	Right	Fall Down	Hypertension, Diabetes	7	90	300	36	Wound infection
12	Female	32	Left	Bicycle Accident	None	2	80	200	36	None
13	Male	40	Right	Fall Down	None	1	85	240	12	None
14	Male	26	Left	Fall Down	None	1	70	150	24	None
15	Male	28	Left	Vehicle Accident	None	1	80	180	24	None
16	Male	34	Right	Fall Down	None	2	85	150	12	None
17	Male	44	Right	Bicycle Accident	None	1	95	180	12	None
18	Female	58	Right	Fall Down	None	3	75	150	12	None
19	Male	42	Left	Fall Down	None	5	80	200	12	None
20	Male	22	Left	Bicycle Accident	None	1	90	130	24	None
21	Male	60	Left	Fall Down	Hypertension	2	85	200	12	None
Mean (SD)		43.1(15.0)				2.4(1.8)	86.(9.2)	203.3(56.6)	20.6(10.1)	

*SD* standard deviation

**Fig. 3:**
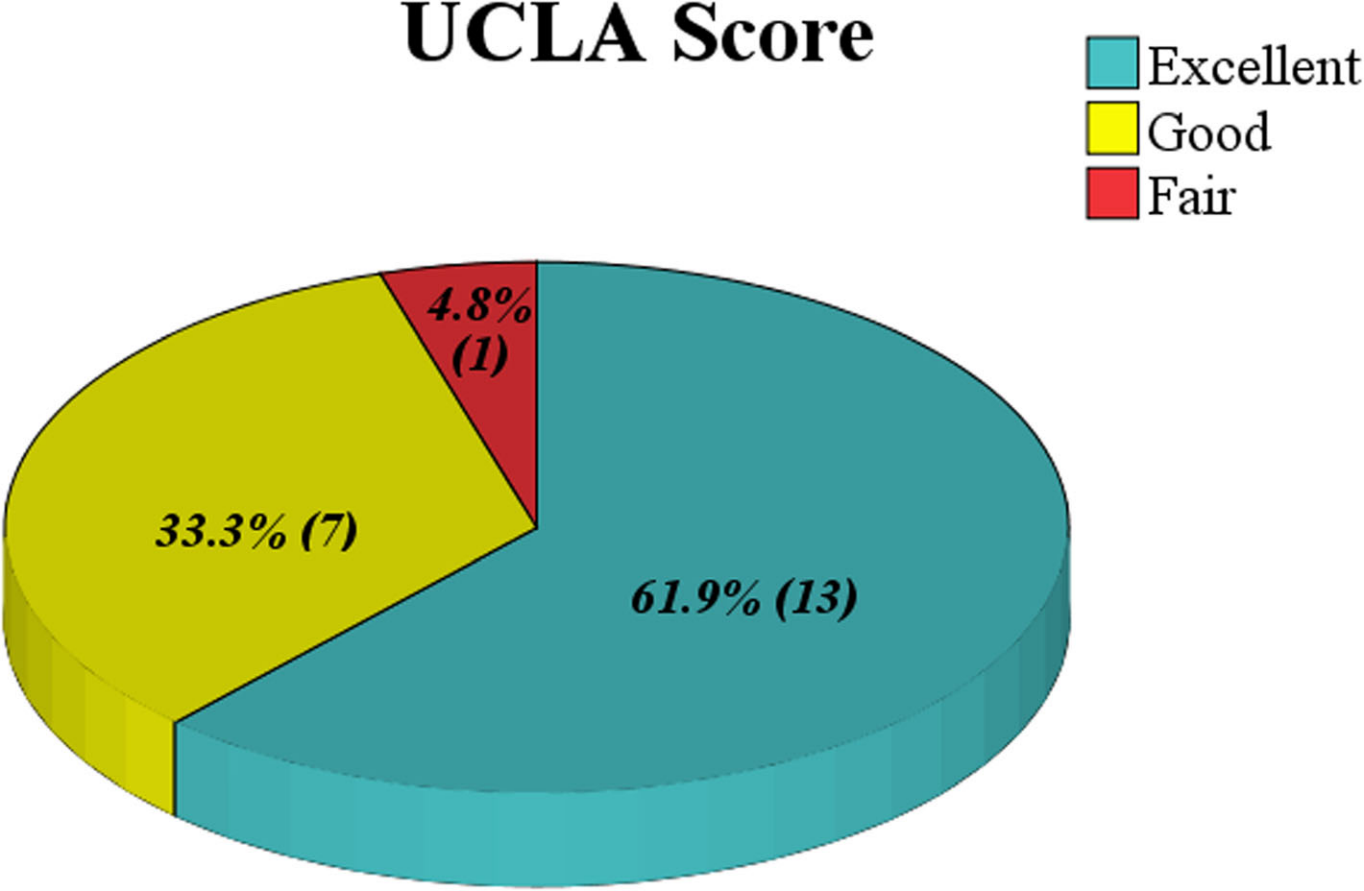
The one-year UCLA score outcome: over 95% of the patients acquired a good or excellent shoulder function recovery within one year

**Fig. 4:**
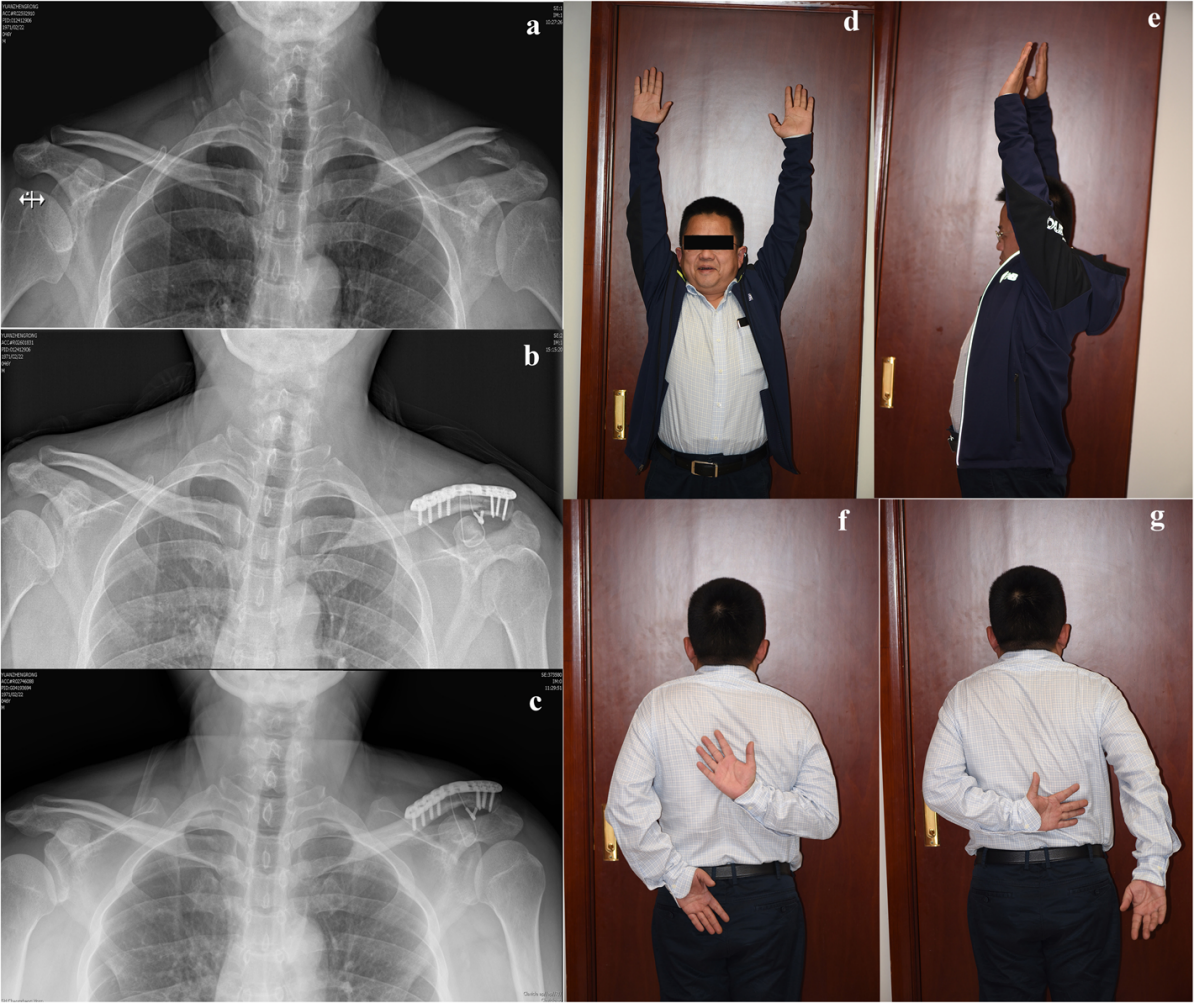
Radiological and functional outcomes of a 46-year-old male with unstable distal clavicle fracture. **a** Pre-operative radiograph showed a Neer type II fracture of the left clavicle. **b** Post-operative radiograph showed an anatomical reduction. **c** At the six-month follow-up, the fracture achieved osseous union and no implant loosening or migration occurred. **d-g** The patient was satisfied with the shoulder function recovery (the Constant score was 98 and the UCLA score was 35 at the one-year follow-up)

At the one-year follow-up, all patients had achieved range of motion to a degree that satisfied criteria for “recovery” for both flexion and rotation. The mean forward flexion was 158.3 ± 5.1°(range150–165°); the external and internal rotation values were 49.6 ± 2.3° (range 45–52°) and 10.3 ± 1.4°(range8–12°) respectively. The mean visual analog scale score was 1.1 ± 1.0 (range0–3). The mean Constant score of the injured shoulder at the one-year follow-up was 94.6 ± 3.3 (range 84–98), and the mean UCLA score was 33.3 ± 2.6 (range 27–35). Twenty patients (accounting for 95.2 %) acquired satisfactory shoulder function within one year according to the UCLA scoring system (including 13 patients with excellent function and 7 patients with good function) (Table 2; Figures 3 and 4).

**Table 2:** Functional outcome at One-year Follow-up

	ROM			VAS	Constant	UCLA
	Forward Flexion	External Rotation	Internal Rotation			
Mean	158.3	49.6	10.3	1.1	94.6	33.3
SD	5.1	2.3	1.4	1.0	3.3	2.6
Max	165	52	12	3	98	35
Min	150	45	8	0	84	27

*ROM* range of motion; *VAS *visual analogue scale; *UCLA* University of California, Los Angeles; *SD* standard deviation; *Max* maximum; *Min* minimum

Only one patient experienced a complication during follow-up. A 55-year-old male was diagnosed with a surgical site infection two weeks postoperatively. The infection was limited to superficial tissue, and was treated successfully with dressing changing and antibiotics. No implant loosening or other complications were found during his subsequent follow-ups. Two patients received another operation for implant removal because of hardware prominence at one year after surgery. For both of these cases, the distal clavicle remained in the anatomical position after removal of the implants.

## Discussion

Surgical treatment is recommended for Neer type II distal clavicle fractures due to the high rate of nonunion and complications associated with conservative treatment [2, 4]. Although some modified classification and a variety of surgical strategies have been proposed in recent studies, the preferred method for treating Neer type II distal clavicle fractures remains controversial [16, 17]. Hook plate fixation has been demonstrated as an effective treatment for management of unstable distal clavicle fracture [18]. This plate prevents superior translation of the medial clavicle while also providing stable fixation of the fracture. However, the relatively high complication rate, as well as the need for a second operation to remove the implant, have limited its utility [19, 20].

The use of a precontoured locking plate has been reported as another effective treatment [7–9]. The specific contoured design allows for additional numbers of locking screws to be inserted into the distal fragment, which can reinforce the fracture fixation. However, for comminuted fracture with poor bone quality, locking plate fixation alone may be insufficient for maintaining stability [21]. Given that the coracoclavicular ligament is known to function in providing stability to the medial fracture fragment, especially in the vertical direction, a CC stabilization technique is necessary [10, 22]. CC screw fixation is a classic method for CC stabilization that has been widely used, yet because this technique employs rigid fixation for the CC ligament, subtle motion of the AC joint is limited [23]. As the subtle motion of AC joint is part of normal shoulder girdle function, an ideal method for CC stabilization should be able to both sustain AC joint stability while also enabling subtle motion [24].

Titanium cables have excellent biomechanical properties and can provide over 1000 N bearing force for reconstructing the CC ligament [25]. The elastic characteristic of titanium cables also meets the requirement for enabling subtle AC joint motion. Ye T et al. [15] successfully used this method for management of acute AC joint dislocation. Li Y et al. [25] used a titanium cable for suspensory CC ligament fixation in treating Neer type II distal clavicle fractures, and reported good clinical outcomes. But in our experience, when dealing with comminuted unstable distal clavicle fractures, use of a titanium cable alone may not be able to maintain the stability of the fracture. Thus, we envisioned a combined surgical strategy: a distal clavicle anatomical locking plate used alongside a titanium cable. For management of unstable distal clavicle fractures, the locking plate provides a rigid fixation to the fracture, while the titanium cable provides an elastic fixation between the clavicle and coracoid. As fracture fixation is reinforced and the subtle motion of AC joint can be retained, this combined surgical strategy should offer good clinical outcomes. Indeed, all patients in the present study achieved osseous union and acquired satisfactory shoulder function, supporting that this combined surgical strategy may represent a reliable choice for orthopedic surgeons to manage unstable distal clavicle fractures.

In recent years, use of a locking plate combined with some kind of CC stabilization technique has been reported for treating unstable distal clavicle fractures [7, 10–14]. Rieser et al. [10] conducted a biomechanical study to evaluate the combination of a locking plate and CC reconstruction in the management of unstable distal clavicle fracture. They reported that improved fracture stability was achieved via combination of CC reconstruction and a distal clavicle locking plate compared to either intervention alone. There are similar findings from other clinical studies investigating use of a locking plate and CC ligament fixation in combination [7, 11–14]. Andersen et al. [7] described use of a pre-contoured superior locking plate in 20 patients with distal clavicle fractures. Nine of these 20 patients were also treated with either suture cerclage or CC screw fixation in addition to the plate. No fixation failure or complication related to the implants was observed during their follow-up period. However, CC screw fixation may not be optimal because it limits subtle motion of the AC joint. Instead, an elastic fixation method may be more appropriate for CC stabilization [26]. Johnston et al. [13]. Proposed a method using a suture button in addition to a locking plate. All patients in that study achieved bony union and satisfactory shoulder function recovery, which suggests that the suture button technique could act as a useful method for CC stabilization. However, the suture button technique may be more technically demanding for orthopedic surgeons, as a 2.4mm hole needs to be drilled from the distal clavicle into the base of the coracoid in order to apply the device [27]. In our study, we used a titanium cable instead of a suture button. By using a tensioner instrument, the titanium cable loop can be easily tightened and secured, making this technique much simpler and more reliable.

We compared our findings in the present study with results from six similar recent studies (Table 3). For this comparison, all patients had to be treated with either a locking T-plate or a contoured locking plate designed for the distal clavicle, and relevant studies included CC ligament fixation methods including a CC screw, suture cerclage, and a suture button. A total of 67 patients achieved osseous union (the union rate is 98.5 % overall); only one patient had an infected nonunion (reported by Andersen et al. [7]), which was subsequently treated with irrigation, debridement, hardware removal, and distal clavicle excision. A total of 10 complications were reported in the examined studies, including three implant irritations, three coracoclavicular ligaments calcifications, two infections, one peri-implant fracture, and one malreduction. In our study, only one patient suffered a complication (surgical site infection) and this was treated successfully with dress changing and antibiotics. In addition, no osteolysis or bony arrosion was detected during the follow-up. This may be due to the fact that the titanium cable acts as an elastic fixation device, so the normal subtle motion of the AC joint was barely interfered. In terms of union rate and shoulder functional outcomes, our results are similar with the examined studies.

**Table 3:** The retrospective series that used distal locking plates combined with CC ligament fixation are compared

Author	Number of patients	Mean follow-up	Fixation method	Union rate	Functional outcome	Complications	Implant removal
Kalamaras et al. [8]	9	13.6 months	1.Synthes distal radius LCP (9/9) 2. Suture cerclage (6/9)	100 %	Average Constant 96	Wound infection (1) Malreduction (1)	0
Herrmann et al. [12]	7	8.3 months	3.5 mm locking T-plate with suture cerclage	100 %	Average Constant 93.3 Average DASH 15.3	None	2
Andersen et al. [7]	20	30.7 months	1. Locking plate (20/20) 2. suture cerclage (9/20) 3. CC screw (2/20)	94 %	Average ASES 79.0	Infected nonunion (1) Peri-implant fracture (1)	4
Schliemann et al. [14]	14	38 months	3.5 mm locking plate with suture button	100 %	Average Constant 93.5	implant irritation (3) CC ligaments calcifications (3)	7
Johnston et al. [13]	6	15.6 months	Distal clavicle locking plate with suture button	100 %	Average ASES 97.97	None	1
Lei Han et al. [11]	12	26.3 months	Anatomical locking plate with suture anchor augmentation	100 %	Average Constant 94 Average DASH 10.4	None	NR
Fan Zhang et al. (present study)	21	20.6 months	Distal clavicle anatomical locking plate with titanium Cable	100 %	Average Constant 94.6 Average UCLA 33.3	Wound infection (1)	2

*CC *coracoclavicular; *ASES* American Shoulder and Elbow Surgeons; *DASH* Disabilities of the Arm, Shoulder and Hand; *NR* not reported

Our study is subject to several limitations. First, this is a retrospective series study with limited cases, which restricts confidence for extrapolating the likelihood of success more widely. Second, lacking a control group, it is difficult to interpret from our study whether the additional CC ligament fixation conferred any extra benefit to patients. Finally, the mean follow-up period of 20.6 months may be not long enough to supporting definitive conclusions about final outcomes and/or long-term complications.

## Conclusions

In conclusion, using a distal clavicle locking plate combined with a titanium cable in the management of Neer type II fractures results in a high union rate, a low complication rate, and good shoulder function. By providing rigid fixation to the fracture site and elastic fixation to the CC ligament, the fixation becomes reinforced while preserving the capacity for subtle motion of the AC joint. Our study supports that, for patients with unstable distal clavicle fractures, this modified combined surgical strategy can be regarded as an apparently reliable treatment. Despite this satisfactory result, the present evidence is insufficient to judge if this new combined surgical strategy is superior to others. Considering the limitations of this study, a prospective cohort study comparing the combined surgical strategy we explored against other surgical methods will still be needed to provide stronger evidence in support of this treatment.

## Data Availability

The datasets during and/or analyzed during the current study are available from the corresponding author on reasonable request.

## Abbreviations

UCLA: University of California, Los Angeles
AC: Acromioclavicular
CC: Coracoclavicular
VAS: The visual analog scale
